# Atraumatic Toe Pain: Acute Calcific Periarthritis

**DOI:** 10.7759/cureus.85479

**Published:** 2025-06-06

**Authors:** Lindsay Mello, Adam Lindsay, Timothy Herbst, Meghan Herbst

**Affiliations:** 1 Department of Emergency Medicine, University of Connecticut School of Medicine, Farmington, USA; 2 Department of Orthopedic Surgery, University of Connecticut School of Medicine, Farmington, USA; 3 Department of Radiology, University of Connecticut School of Medicine, Farmington, USA

**Keywords:** acute calcific periarthritis, joint disease, joint pain, joint swelling, monoarthritis

## Abstract

Acute calcific periarthritis (ACP) is a self-limiting but often underrecognized condition that can closely mimics more serious joint pathologies such as septic arthritis. We present the case of a 43-year-old woman who developed acute pain and swelling of the right second toe after a 4-mile run. Initial evaluation in the emergency department (ED) included point-of-care ultrasound and C-arm imaging, which revealed periarticular calcification at the distal interphalangeal joint. However, the diagnosis of ACP was not made at that time. The patient was treated with nonsteroidal anti-inflammatory medication and referred for outpatient orthopedic evaluation. Despite symptomatic improvement and partial resolution of the calcification on radiographs one week later, the diagnosis remained unclear until two months later, when an orthopedic oncologist identified the condition as ACP.

ACP results from hydroxyapatite crystal deposition in periarticular tissues and is most commonly seen in the shoulder but may also involve the hands, feet, and other joints. It presents with acute monoarticular pain, swelling, erythema, and limited range of motion. These features may overlap with crystal arthropathies, infection, and intra-articular injury. Radiographs and ultrasound are useful tools for identifying characteristic calcifications and ruling out alternative etiologies. Recognition of ACP in the ED is important to avoid unnecessary interventions, consultations, and diagnostic delays. This case highlights the clinical and imaging features of ACP and emphasizes the importance of including it in the differential diagnosis of acute monoarticular joint pain.

## Introduction

Musculoskeletal complaints account for more than 25% of all emergency department (ED) visits, with a wide range of etiologies [1]. For acute joint pain specifically, diagnostic considerations include infection, reactive arthritis, crystal arthropathy, and intra-articular injury [2]. Differentiating serious conditions that can lead to joint destruction or systemic complications, such as septic arthritis, from benign, self-limiting arthropathies relies on a combination of history, physical examination, and imaging evaluation [3].

Acute calcific periarthritis (ACP) is an uncommon and often underrecognized entity caused by periarticular deposition of hydroxyapatite crystals [4-6]. It most frequently affects the shoulder but can occur in other joints, including the hip, wrist, and fingers, and has been rarely reported in the toes [7]. The acute presentation typically includes localized pain, swelling, and functional impairment, sometimes accompanied by systemic symptoms, making it difficult to distinguish from infectious or inflammatory arthritides without appropriate imaging [8,9]. Misdiagnosis may result in unnecessary interventions, referrals, and delayed symptom resolution [10].

We present a case of ACP in an atypical location that was initially misdiagnosed in the ED, leading to multiple specialist evaluations and a delayed definitive diagnosis. The case highlights the need for increased awareness of ACP as a benign, self-limited cause of acute monoarthritis.

## Case presentation

A 43-year-old woman with no significant past medical history presented with four days of right second toe pain. The pain began while she was at rest, approximately one hour after her daily 4-mile run, and worsened over the next 24 hours, accompanied by toe swelling, causing her to limp. She denied fever, rash, fatigue, other joint involvement, recent injury, and gastrointestinal or genitourinary symptoms. Physical examination revealed a diffusely swollen second toe with good capillary refill, restricted range of motion, and plantar induration without fluctuance or obvious deformity.

**Figure 1:**
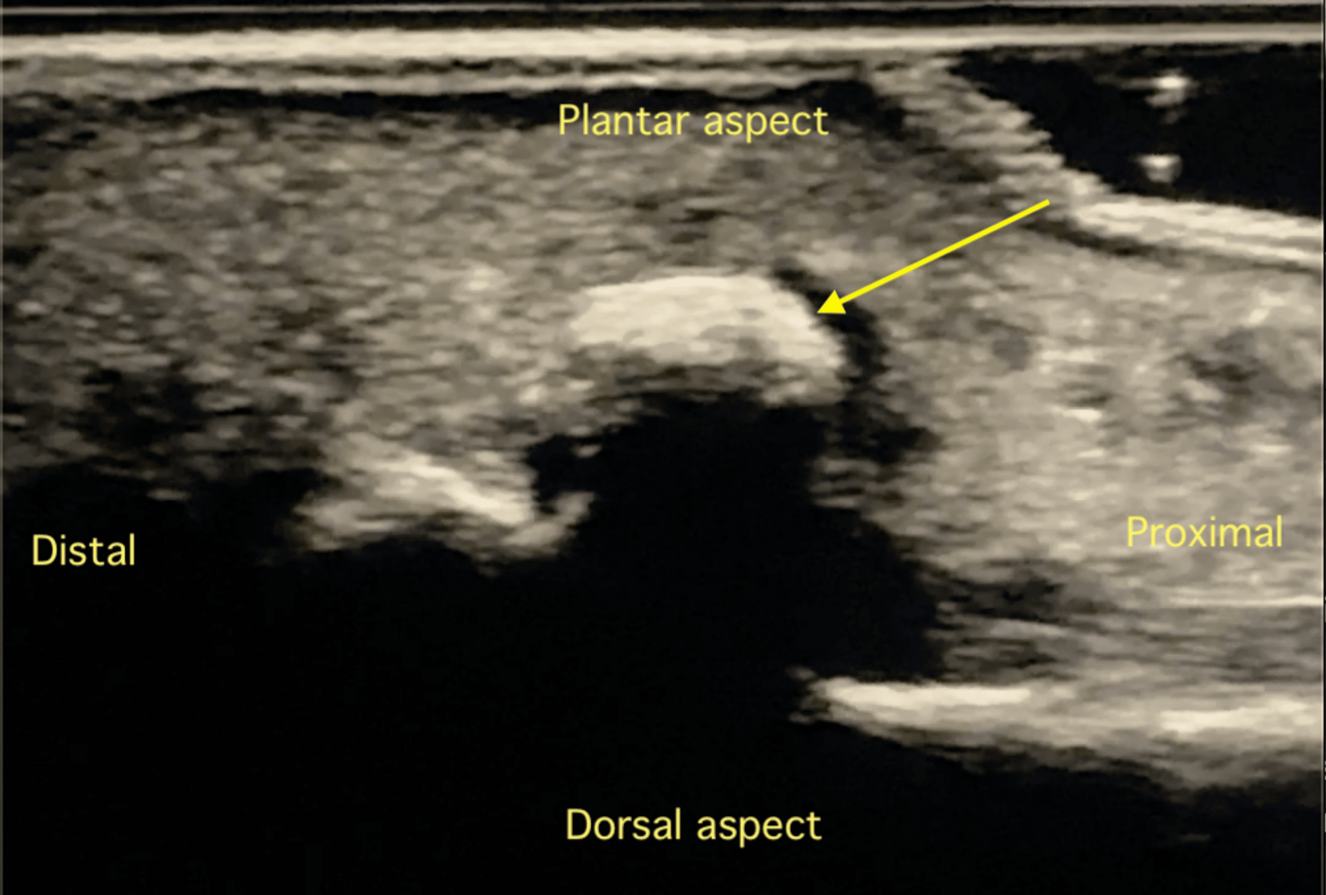
Ultrasound image using a linear transducer of extra-articular calcification (arrow) at the plantar aspect of distal interphalangeal joint

**Figure 2:**
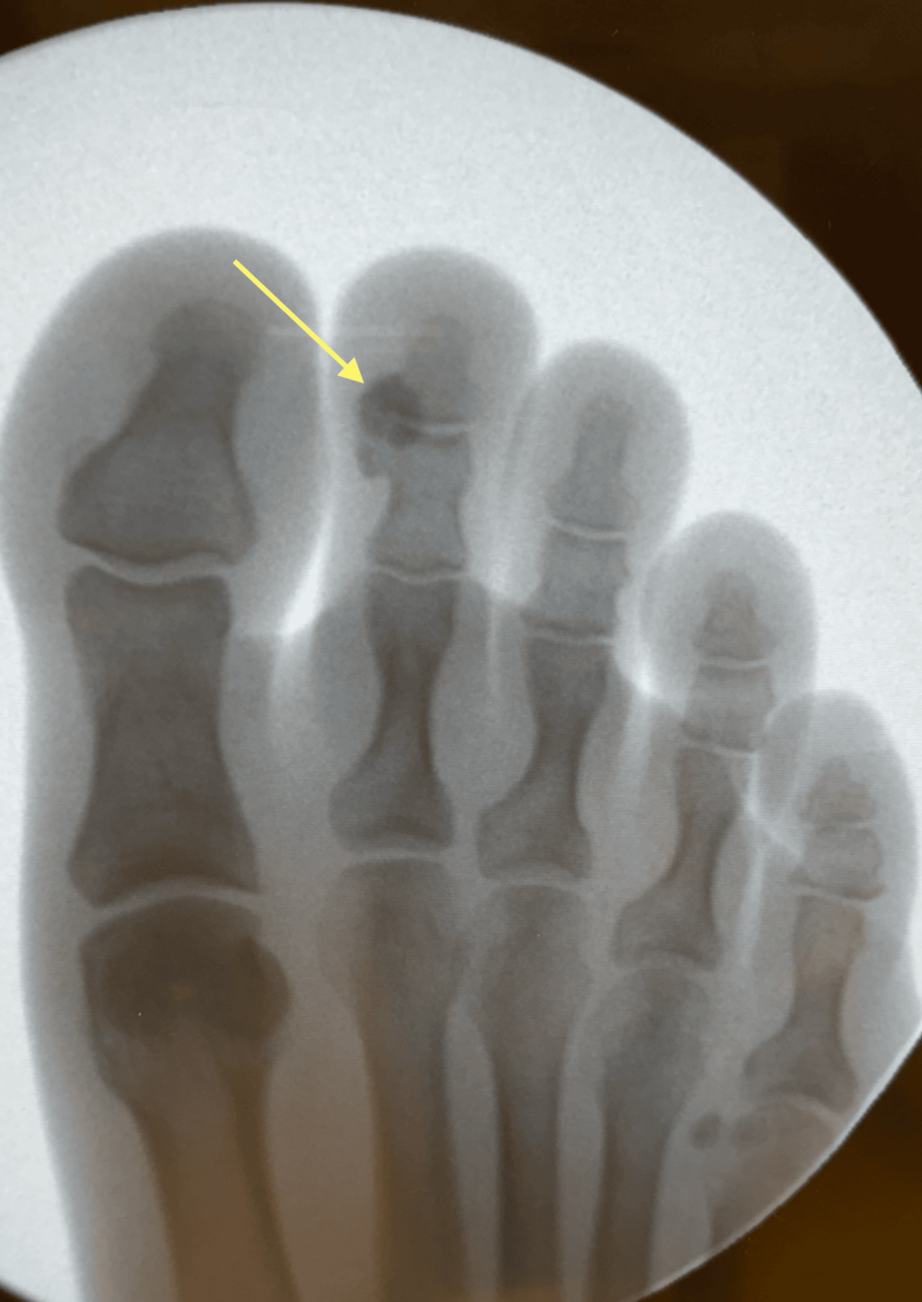
C-arm image of calcium deposit (arrow) at the second toe distal interphalangeal joint

The differential included fractures, sesamoiditis, sprains, tendonitis, crystal arthropathy, septic arthritis, or abscesses. At her initial ED visit, ultrasound revealed a calcification at the distal interphalangeal joint (Figure 1), confirmed by C-arm imaging (Figure 2). However, this finding was not recognized as consistent with ACP at the time.

**Figure 3:**
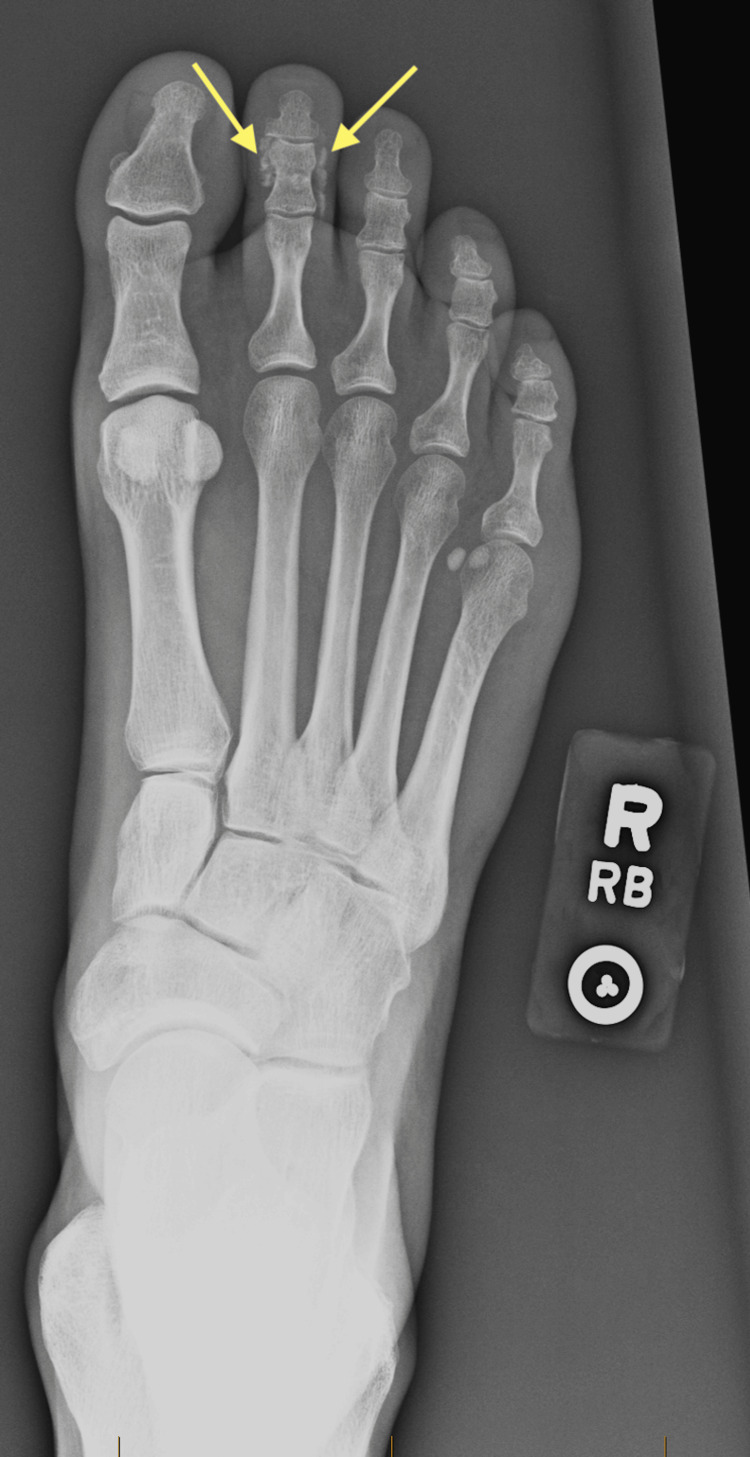
X-ray confirming periarticular calcification (arrows)

She was advised to take ibuprofen for pain, to limit weight-bearing on the affected foot for comfort, and was referred to an orthopedic foot specialist for further evaluation. At her follow-up one week later, foot radiography demonstrated partial resolution of the periarticular calcification (Figure 3). Her pain had largely resolved, but due to diagnostic uncertainty, the orthopedic specialist referred her to an orthopedic oncologist, to whom she presented two months later and was ultimately diagnosed with ACP.

## Discussion

ACP is an often misdiagnosed mono-periarticular process involving dystrophic calcium hydroxyapatite deposition [5]. It appears as distinct periarticular densities within the joint capsule that lack evidence of ossification. Deposits in adjacent tendinous or ligamentous tissues may occur as well [5,11].

Patients with ACP typically present with the sudden onset of pain, localized swelling, erythema, tenderness, and restricted range of motion [12]. The shoulder is the most common joint involved, but occurrence in the hands, fingers, feet, and toes has also been described in the literature [11]. ACP can affect men and women over a wide age range, from 31 to 88 years, in one series of 15 cases [4], and follows trauma or microtrauma in one-third of cases [12]. Some reports suggest women are more commonly affected [4]. ACP is self-limiting, and relapse is uncommon; symptoms typically improve in four to seven days with complete resolution in three to four weeks [11]. Treatment options include local corticosteroid injections, nonsteroidal anti-inflammatory drugs, and simply rest [12].

The pathophysiology of ACP is not well understood. One theory is that mechanical, metabolic, or other factors decrease blood flow, leading to local hypoxia at the critical areas of the tendon, ligament, or capsule, resulting in calcium deposition [12]. The condition progresses through four described phases: 1) precalcific: tendon hypoxia leads to fibrocartilage formation; 2) calcific: chondrocyte-mediated calcium apatite deposition; 3) resorptive: lymphocyte and giant cell infiltration form a granuloma; and 4) postcalcific: capillary growth and collagen fiber remodeling [9,13].

Imaging for ACP diagnosis ranges from plain radiograph to computed tomography and magnetic resonance imaging [8,9,12]. On radiographs, deposits of ACP are distinct, homogenous periarticular densities with no ossification. Calcium deposits may involve the joint capsule, tendon at the insertion site, or ligaments, but they do not occur within the joint itself. This feature can help differentiate ACP from other diagnoses. The morphology and mineralization may change over time, and most calcifications will resolve [9].

The use of ultrasound can further differentiate ACP from other monoarticular joint pain pathology [8,14]. Ultrasound can identify the calcification, provide information on the soft or hard nature of the calcification, and differentiate its phase using localized Doppler flow and probe pressure tenderness [14]. It may additionally identify an echogenic focus within an abnormally thickened and hypoechoic ligament or tendon representing calcification [14]. Color Doppler may demonstrate capsular or pericapsular hyperemia [9]. Ultrasound can additionally rule out crystal arthropathies and septic arthritis, given its high sensitivity for joint effusions [14]. If ultrasound is not definitive, a follow-up radiograph can help differentiate alternative pathologies from ACP [9,14].

ACP can mimic gout, pseudogout, and septic arthritis, with up to 70% of cases misdiagnosed, leading to unnecessary imaging, procedures, antibiotics, and other medications [5,15]. Table 1 lists common etiologies for acute monoarticular pain, diagnostic modalities, and recommended management. Calcifications seen in gout are associated with periarticular erosions and typically present in the intermediate or late stages as a tophus, whereas calcifications associated with ACP present acutely and are not associated with erosions [9,14]. In pseudogout, calcium pyrophosphate dihydrate crystals are seen in fibrous or hyaline cartilage, unlike ACP [9]. Septic arthritis is associated with a joint effusion, unlike ACP [2].

**Table 1:** Differential diagnosis of acute monoarticular joint pain NSAID: nonsteroidal anti-inflammatory drug; ROM: range of motion; MTP: metatarsophalangeal; CRP: C-reactive protein; ESR: erythrocyte sedimentation rate

Condition	Symptoms	Diagnosis	Intervention
Acute calcific periarthritis	Localized swelling, erythema, tenderness, and restricted ROM; usually involves shoulder and rare recurrence	Radiograph: homogeneous periarticular calcification without ossification; ultrasound: no effusion, possible hyperemia	NSAIDs, corticosteroid injection, rest, and self-limited in three to four weeks
Gout	Intense pain, swelling, and erythema; usually involves first MTP joint and common recurrence	Synovial fluid: needle-shaped, negatively birefringent crystals; serum urate ↑; radiograph: tophi, erosions; ultrasound: effusion, tophi, and double contour sign	NSAIDs, colchicine, steroids, and long-term urate-lowering therapy
Pseudogout	Can be subacute, often in knees/wrists; swelling, stiffness, and common recurrence	Synovial fluid: rhomboid and weakly birefringent crystals; radiograph: chondrocalcinosis; ultrasound: effusion, chondrocalcinosis	NSAIDs, colchicine, corticosteroids, and arthrocentesis
Septic arthritis	Systemic signs: fever, malaise, erythema, and limited ROM	Synovial fluid: purulent, high WBC, Gram stain/culture positive; Labs: ↑ CRP/ESR; ultrasound: effusion	Intravenous antibiotics, arthrocentesis, and admission
Intra-articular injury (e.g., fracture)	Follows trauma, swelling, bruising, deformity, possible crepitus, or inability to bear weight	Radiograph: fracture line, joint space involvement	Immobilization, possible surgery, and pain control
Sesamoiditis	Chronic or subacute pain under first MTP joint; worse pain with activity or pressure	Clinical: focal tenderness over sesamoids; radiograph: may show fracture or bipartite sesamoids	Activity modification, padding/orthotics, NSAIDs, and rarely steroid injection or surgery

Altogether, a periarticular calcification lacking ossification and absence of an effusion in a patient with a single acutely painful joint that is warm, edematous, and tender with restricted range of motion is consistent with ACP [9,14]. As ACP remains an uncommon diagnosis and the current literature is predominantly composed of case reports, case series, and narrative reviews, key aspects such as misdiagnosis rates, epidemiological data, and clinical significance are not yet well established or fully understood. This case highlights a lack of clinician awareness of ACP in the emergency medicine setting and underscores the importance of including it in the differential for monoarticular pain, with the use of readily available ultrasound to support timely diagnosis.

## Conclusions

ACP is a benign, self-limiting condition that can present with features that closely resemble more serious pathologies, such as septic arthritis. This overlap in clinical presentation can make accurate diagnosis challenging, especially in emergency settings where timely decision-making is critical. In the case presented, although imaging demonstrated characteristic calcifications, the lack of familiarity with ACP likely contributed to a delay in diagnosis and multiple referrals. Emergency physicians should consider ACP in the setting of monoarticular pain with periarticular calcification (without joint effusion) and leverage ultrasound and radiographs for early recognition. By incorporating ACP into the differential diagnosis when appropriate, emergency physicians can improve diagnostic efficiency, reduce patient anxiety, and avoid unnecessary tests and specialist consultations.
